# Case Report: Prosthetic revision due to aseptic loosening following total knee arthroplasty: a clinical management and pathological mechanism investigation

**DOI:** 10.3389/fsurg.2025.1710878

**Published:** 2026-01-12

**Authors:** Jun Li, He Shang, Tao Ma, Tianxiang Yang, Yi Wang, Xueqi Liu, Xing He, Yumei Ding, Jinpeng Liang, Yinbin Wang, Desheng Chen

**Affiliations:** 1 Third Clinical Medical College of Ningxia Medical University, Yinchuan, China; 2 Department of Joint Surgery, Ningxia Hui Autonomous Region People’s Hospital (Affiliated Hospital of Ningxia Medical University), Yinchuan, China

**Keywords:** aseptic loosening, autophagy dysregulation, immunohistochemistry, synovial inflammation, total knee arthroplasty revision

## Abstract

**Background:**

Total joint arthroplasty is an effective treatment for end-stage joint diseases, with approximately 1.5 million procedures performed globally annually and a 25%–30% annual growth rate in China. However, 10%–15% of patients develop prosthetic loosening or subsidence within 15–20 years postoperatively, predominantly due to aseptic loosening (incidence >10%) caused by wear particle-induced aseptic inflammatory osteolysis. The role of autophagy in this pathogenesis remains incompletely understood.

**Methods:**

A 61-year-old female patient developed aseptic loosening 11 months after left total knee arthroplasty. Comprehensive management included preoperative screening (including synovial cell count, differential, and alpha-defensin detection), revision surgery (debridement of necrotic/inflammatory tissue/residual cement and implantation of a new prosthesis with vancomycin-impregnated cement), synovial HE staining, quantitative immunohistochemistry (IHC; Ki67, CD3, CD20, CD68, P62, LC3II, and Beclin1), and postoperative rehabilitation.

**Results:**

Postoperatively, pain was relieved: the patient ambulated with crutches at 3 days, achieved 90° knee flexion at 1 week, and full pain-free weight-bearing (110° flexion) at 2 months. Postoperative infection markers (C-reactive protein and erythrocyte sedimentation rate) were temporarily elevated due to surgical trauma and returned to normal during follow-up. Imaging showed a stable prosthesis without infection or recurrent loosening. Synovial HE staining revealed extensive inflammatory infiltration; quantitative IHC showed high expression of inflammatory markers and low expression of autophagy-related markers. Clinical outcomes were favorable with validated patient-reported outcome measures (Knee injury and Osteoarthritis Outcome Score: 85 points; Western Ontario and McMaster Universities Osteoarthritis Index score: 20 points) at 6 months post-revision.

**Conclusion:**

The integrated protocol effectively treated aseptic loosening. Wear particle-induced chronic synovitis and altered autophagy-related marker expression may be involved in the pathogenesis, providing preliminary clinical and pathological evidence for further research.

## 1 Introduction

Total joint arthroplasty (TJA) is the gold standard for treating end-stage joint diseases such as osteoarthritis and rheumatoid arthritis. Globally, approximately 1.5 million TJA procedures are performed annually, with a 25%–30% annual growth rate in China (1). However, aseptic loosening is a major long-term complication, affecting 10%–15% of patients within 15–20 years postoperatively, primarily due to wear particle-induced aseptic inflammatory osteolysis (incidence >10%) (1). Macrophages phagocytose wear particles (e.g., polyethylene and titanium) and release pro-inflammatory cytokines (TNF-α, IL-1, IL-6), promoting chronic inflammation, osteoclast differentiation, and bone metabolism imbalance. Autophagy, a cellular homeostasis mechanism, regulates osteoblast and osteoclast function, and its dysregulation may contribute to osteolysis. Inhibition of autophagy has been shown to suppress osteoclast formation and reduce osteolysis-related gene expression (2). However, the role of autophagy in early aseptic loosening (<1 year postoperatively) remains unclear. Herein, we report a case of early aseptic loosening treated with revision surgery, supplemented by clinical, pathological, and follow-up data, in accordance with the CAse REport (CARE) guidelines.

## 2 Case presentation

A 61-year-old female patient underwent left total knee arthroplasty for knee osteoarthritis 11 months prior (primary and revision surgeries were performed by the same attending surgeon: Desheng Chen). The primary prosthesis was manufactured by Zimmer Biomet (model: Persona® Total Knee System, USA). She developed left lower limb weakness and pain during ambulation 11 months postoperatively, which were relieved by rest, without numbness, sensory abnormalities, or systemic symptoms.

### 2.1 Preoperative evaluations

**Figure 1:**
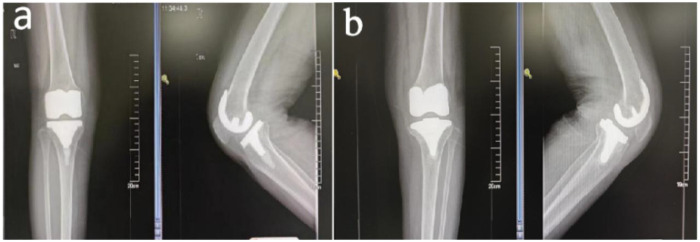
Preoperative anteroposterior and lateral X-rays of both knees: **(a)** right knee, **(b)** left knee.

Imaging: Anteroposterior and lateral radiographs of the left knee showed >2 mm radiolucent lines around the prosthesis with progressive widening, consistent with prosthetic loosening (3) (Figure 1).

Laboratory tests: White blood cells (WBC; 5.14 × 10⁹/L), C-reactive protein (CRP; 1.37 mg/L), erythrocyte sedimentation rate (ESR; 11 mm/h), and osteocalcin (17.27 ng/mL) were within normal ranges (Table 1).

**Table 1:** Preoperative laboratory tests.

Inspection items	Result	Normal range	Significance
White blood cells	5.14 × 10⁹/L	3.5–9.5 × 10⁹/L	No acute infection
C-reactive protein	1.37 mg/L	0–6 mg/L	Inflammatory markers normal
Erythrocyte sedimentation rate	11 mm/h	0–38 mm/h	No chronic inflammation
Osteocalcin	17.27 ng/mL	11–48 ng/mL	Normal bone metabolism
Synovial cell count	80 cells/μL	<200 cells/μL	No evidence of PJI
Synovial neutrophil differential	25%	<70%	No evidence of PJI
Alpha-defensin	Negative	Negative	Excludes PJI

Infection exclusion: Synovial fluid analysis revealed a synovial cell count of 80 cells/μL and a neutrophil differential of 25% and was negative for alpha-defensin, excluding periprosthetic joint infection (PJI). PCR analysis of synovial fluid was not performed due to negative basic infection markers and absence of PJI clinical manifestations (4).

Physical examination: The patient’s left knee had a 10 cm surgical scar (no redness/ulceration), mild tenderness at the anteromedial joint line, restricted patellar mobility, a positive mediolateral stress test, and normal muscle tone/strength (Grade 5). The right knee was unremarkable.

### 2.2 Differential diagnosis

PJI: Excluded by normal infection markers (WBC, CRP, and ESR), negative synovial alpha-defensin, low synovial cell count, and absence of systemic inflammatory symptoms.

Prosthetic dislocation/fracture: Ruled out by radiographs showing no displacement or bony lesions.

Soft tissue injury: Unlikely due to pain correlation with ambulation and radiological loosening signs.

### 2.3 Patient perspective

The patient reported significant interference with daily activities (e.g., climbing stairs and shopping) due to pain and weakness. She desired symptom relief and functional recovery, and provided written informed consent for revision surgery and publication of clinical data.

### 2.4 Timeline

**Figure 2:**
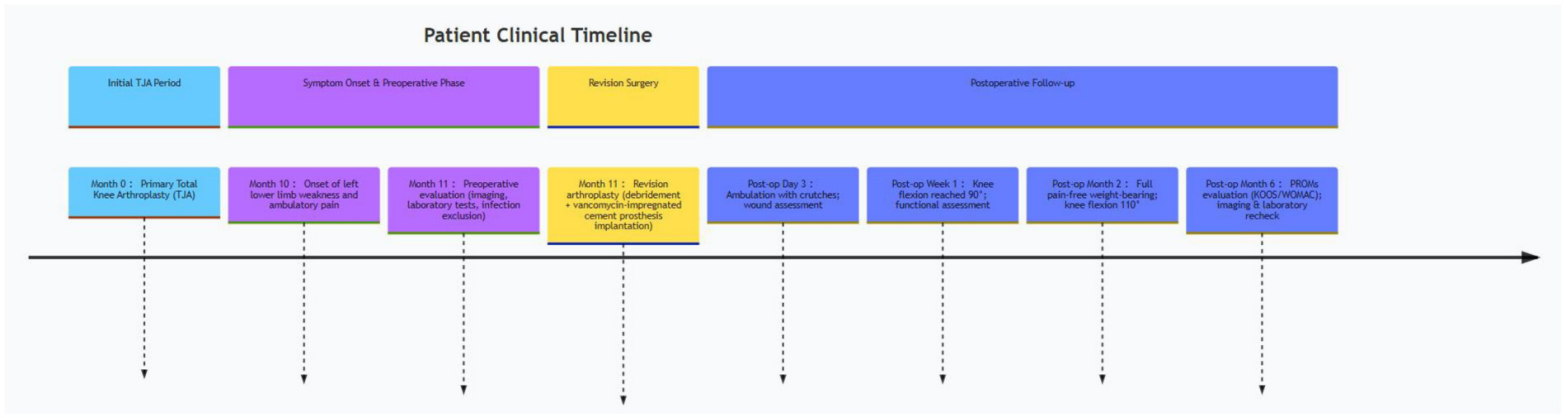
Patient timeline demonstrating key clinical events.

Figure 2 illustrates the patient’s timeline as follows: (1) initial TJA (month 0); (2) onset of symptoms (month 10); (3) preoperative evaluation (month 11); (4) revision surgery (month 11); (5) 3-day, 1-week, 2-month, and 6-month follow-ups.

## 3 Treatment

### 3.1 Surgical treatment

**Figure 3:**
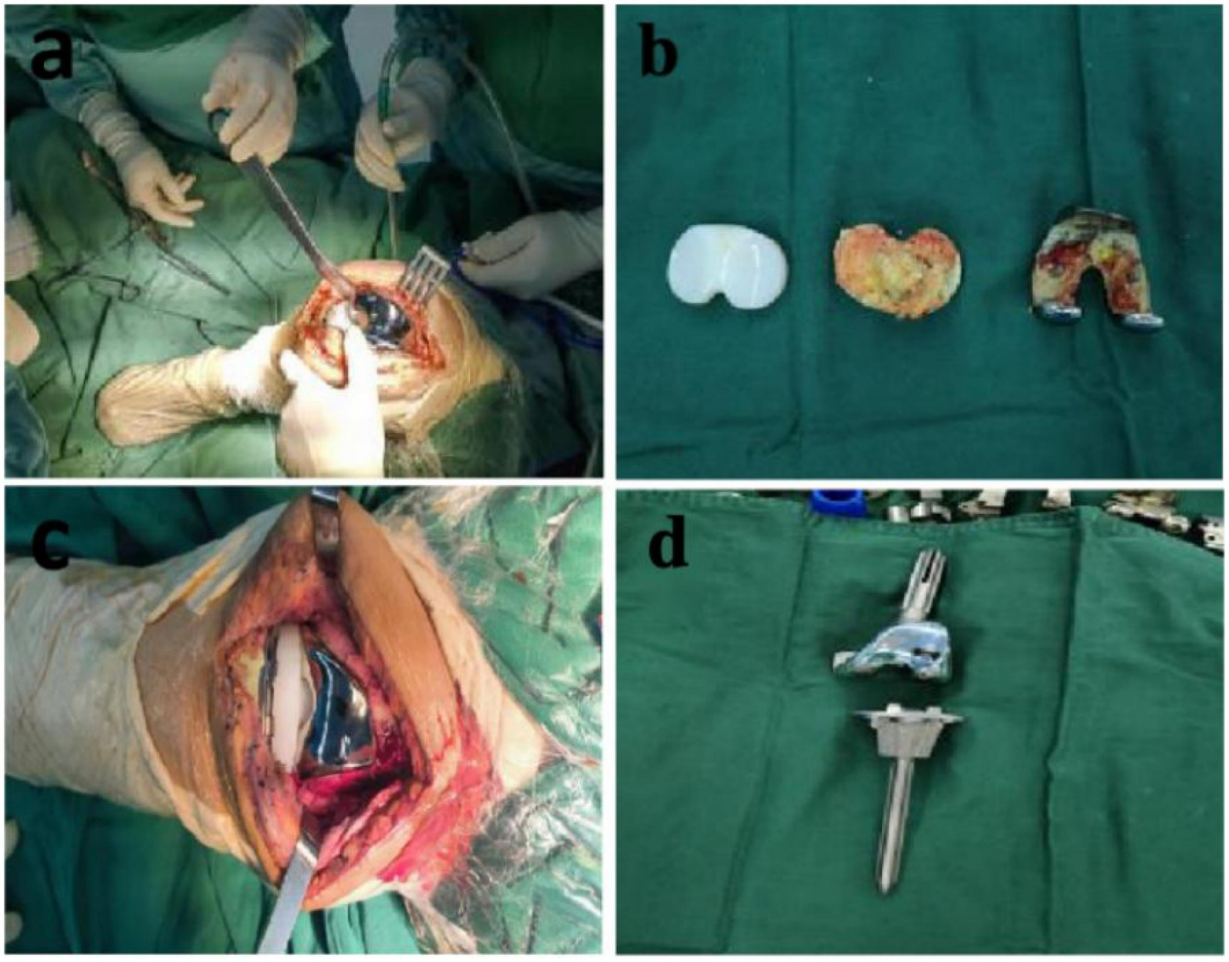
Surgical images: **(a)** and **(c)** intraoperative knee joint images, **(b)** prosthesis with aseptic loosening, (**d**) extended-stem prosthesis for revision arthroplasty.

Under general anesthesia, the patient was placed in the supine position with a pneumatic tourniquet (240 mmHg). The previous anterior midline incision was used to expose the joint. Approximately 5 mL of bloody synovial fluid was aspirated for bacterial culture (negative). Debridement of necrotic tissue, inflammatory granulation tissue, and residual bone cement was performed, followed by irrigation with hydrogen peroxide, normal saline, and povidone-iodine saline (soaked for 30 min). Medullary reaming of the proximal tibia (16 mm) and femoral intercondylar region (12 mm) was performed, and an extended-stem prosthesis (Aikang ACCK, Aikang Medical Co., Ltd., Jinan, Shandong Province, China) was implanted with vancomycin-impregnated cement (80 g cement + 7 g vancomycin) (Figure 3). An additional 3 g of vancomycin was placed in the wound, and a drainage tube was inserted. Intraoperative blood loss was approximately 100 mL (no transfusion).

### 3.2 Postoperative management

Rehabilitation: Passive range-of-motion exercises (3–4 times/day and 10–15 min/session) starting on postoperative day 1, with gradual transition to active exercises. No high-impact activities for 6 months.

Pharmacotherapy: Ketorolac tromethamine (two doses) for acute pain; no antibiotics postoperatively (local vancomycin sufficient for infection prevention).

### 3.3 Follow-up outcomes

Functional recovery: Ambulation with crutches (3 days), 90° knee flexion (1 week), pain-free full weight-bearing with 110° flexion (2 months), and 115° flexion (6 months).

Patient-reported outcome measures (PROMs): Knee injury and Osteoarthritis Outcome Score (KOOS) (85 points, 6 months) and Western Ontario and McMaster Universities Osteoarthritis Index score (20 points, 6 months), indicating significant functional improvement.

**Figure 4:**
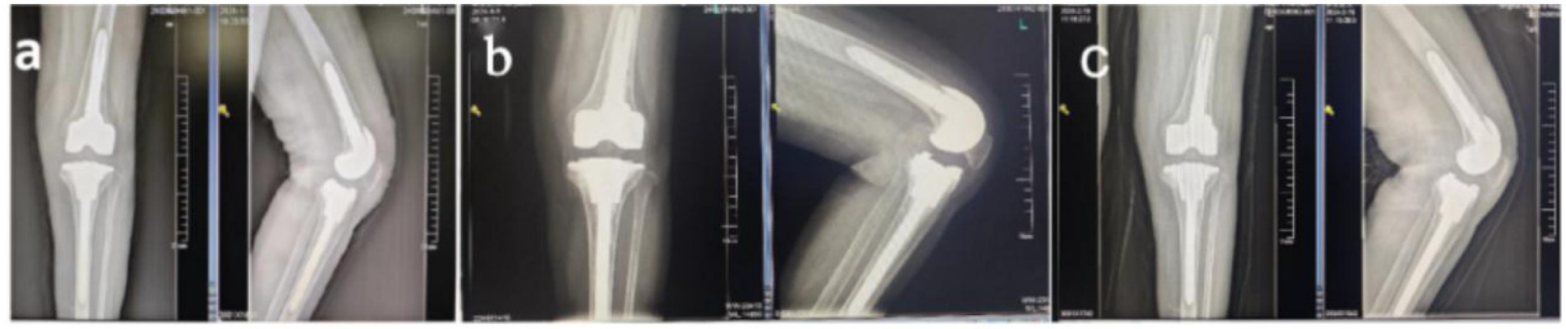
Postoperative images of left knee revision arthroplasty: **(a)** 3 days post-revision, **(b)** 1 month post-revision, **(c)** 6 months post-revision.

Imaging: Postoperative radiographs (at 3 days, 1 month, and 6 months) showed a stable prosthesis without loosening or osteolysis (Kellgren–Lawrence score: Grade 1) (Figure 4).

Laboratory tests: Postoperative CRP (61.39 mg/L) and ESR (68 mm/h) were elevated due to surgical trauma, returning to normal (CRP: 3.2 mg/L; ESR: 15 mm/h) at 6 months. Bacterial/fungal cultures were negative (Table 2).

**Table 2:** Postoperative laboratory tests.

Inspection items	Result (3 days)	Result (6 months)	Normal range	Significance
White blood cells	4.23 × 10⁹/L	5.01 × 10⁹/L	3.5–9.5 × 10⁹/L	No acute infection
C-reactive protein	61.39 mg/L	3.2 mg/L	0–6 mg/L	Transient elevation due to surgical trauma
Erythrocyte sedimentation rate	68 mm/h	15 mm/h	0–38 mm/h	Transient elevation due to surgical trauma
Fungal smear	No mycelium/spores	–	–	No fungal infection
Bacterial culture	Negative	–	–	No bacterial infection

### 3.4 Pathological findings

**Figure 5:**
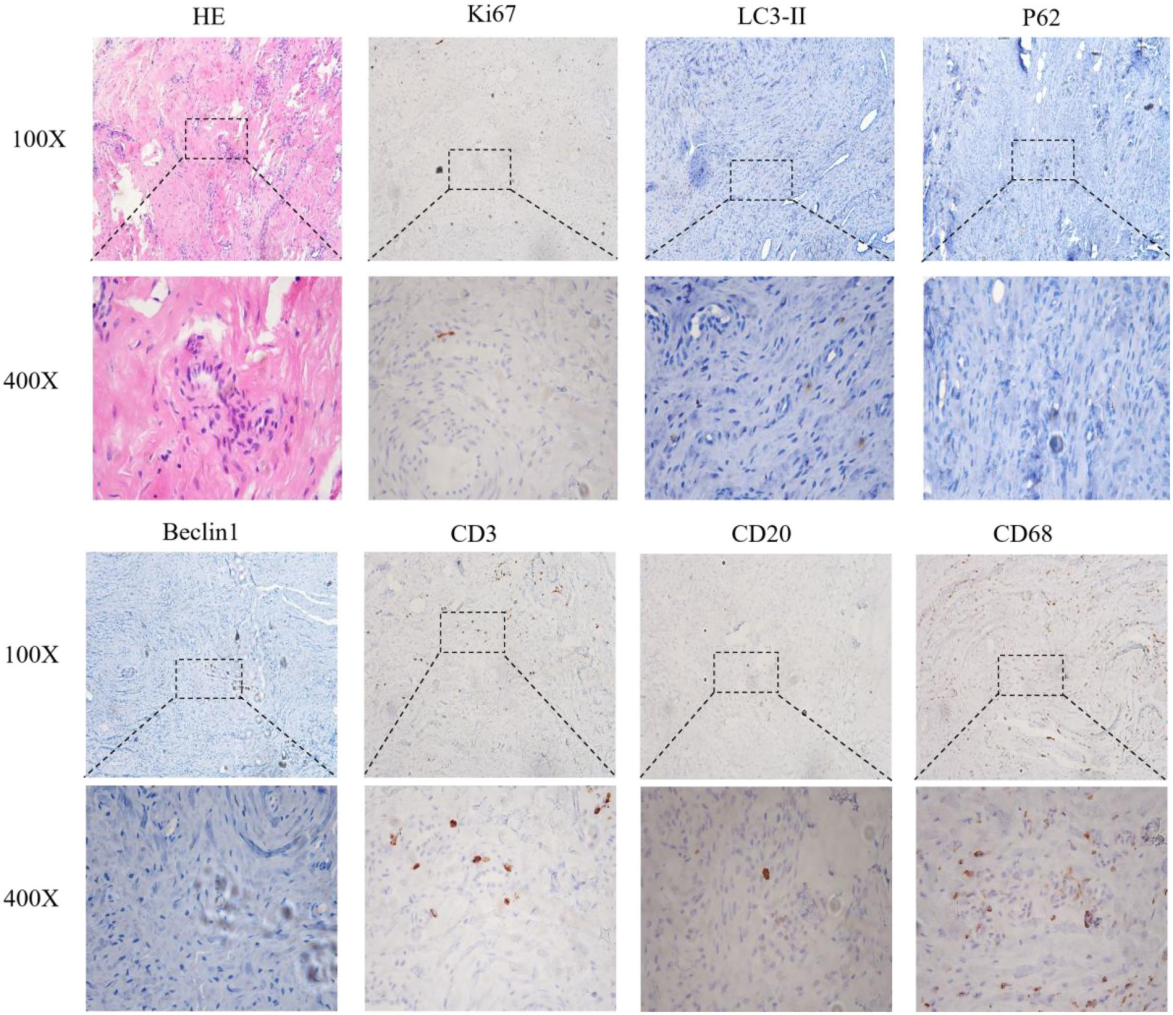
Immunohistochemical staining of synovial specimens from the patient undergoing revision surgery for aseptic loosening of artificial joints.

Synovial HE staining showed extensive inflammatory cell infiltration. Quantitative immunohistochemistry (IHC; positive cell percentage) revealed the following: Ki67 (35%), CD3 (28%), CD20 (15%), CD68 (42%), Beclin1 (12%), LC3II (10%), and P62 (8%) (Figure 5). The high expression of inflammatory markers (CD68, CD3, CD20, and Ki67) indicated active local inflammation, while the low expression of autophagy-related markers (Beclin1, LC3II, and P62) suggested altered autophagy function (no autophagy flux assay was performed, so the mechanistic conclusions are preliminary).

## 4 Discussion

### 4.1 Pathogenesis of implant aseptic loosening and its pathological correlation with this case

The core mechanisms linking the pathogenesis of aseptic prosthesis loosening to the pathological relevance in this case involve wear particle-induced inflammatory responses and abnormal autophagy function. First, the inflammatory response triggered by wear particles is a critical initiation process for prosthesis loosening. Persistent and intense inflammation is recognized as a primary cause of wear particle-induced aseptic osteolysis (5). Particles generated from prosthesis wear, such as titanium or polyethylene particles, can be phagocytosed by macrophages, activating inflammatory signaling pathways that release pro-inflammatory cytokines (e.g., TNF-α and IL-1) and recruit immune cells to accumulate around the prosthesis (6). In this case, HE staining of synovial tissue revealed extensive inflammatory cell infiltration, and the immunohistochemical analysis showed elevated CD68 expression (a macrophage marker). This aligns closely with the literature, highlighting the pivotal role of macrophages in periprosthetic inflammation and pathological bone resorption (7). Concurrently, the expression of CD3 (T-cell marker) and CD20 (B-cell marker) indicates involvement of other immune cells, such as T and B cells, with high Ki67 expression (a proliferation marker), further suggesting active local tissue proliferation that may exacerbate the inflammatory response and osteolytic progression. Second, aberrant autophagy function plays a significant regulatory role in prosthesis loosening. Corpus evidence confirms that autophagy is a key mechanism underlying wear particle-induced osteolysis (1). For instance, inhibiting autophagy—through Atg5 gene knockdown or autophagy inhibitors such as 3-MA and LY294002—reduces osteoclast differentiation and downregulates the expression of osteoclast-related genes (e.g., *TRAP* and *MMP-9*), thereby suppressing bone resorption (8). In this case, the synovial tissue exhibited low expression of autophagy-related markers, with reduced Beclin1 (essential for autophagy initiation) suggesting impaired autophagy start-up, diminished LC3II (an autophagosome marker protein) reflecting decreased autophagosome formation, and low P62 (an autophagic degradation substrate) indirectly indicating inhibited autophagic flux, potentially due to insufficient initiation reducing substrate accumulation. Due to the lack of autophagy flux assays (e.g., chloroquine treatment) and quantitative PCR validation, these results only suggest a potential association between autophagy marker changes and aseptic loosening, requiring further functional studies.

### 4.2 The efficacy of revision surgery and the importance of perioperative management

The successful implementation of revision surgery for aseptic loosening in this case offers valuable clinical insights for the management of prosthesis-related complications. From a surgical perspective, an intraoperative strategy encompassing thorough debridement of necrotic tissue and residual bone cement, combined with antibiotic-loaded cement for infection prophylaxis and meticulous prosthesis implantation, was employed. Specifically, extensive irrigation of the joint cavity using hydrogen peroxide, saline, and povidone-iodine solutions, followed by povidone-iodine immersion, was conducted to effectively remove wear particles and inflammatory mediators, as wear particle-induced inflammation is a key pathological mechanism underlying periprosthetic osteolysis and subsequent loosening (9). This approach aimed to mitigate postoperative infection risks. Additionally, vancomycin was incorporated into the bone cement (7 g per 80 g of cement), supplemented by an extra 3 g placed within the incision to achieve a high localized antibiotic concentration for targeted infection prevention. This method aligns with evidence demonstrating that local antibiotic administration can significantly reduce infection rates after revision procedures (10). Postoperatively, no infections occurred, confirming the efficacy of the infection control protocol. Regarding postoperative recovery, the patient achieved ambulation with crutches within 3 days, achieved 90° knee flexion within 1 week, and progressed to full ambulation without aids with 110° knee flexion by 2 months. This recovery trajectory was notably faster than conventional reports of full ambulation typically requiring 3–4 weeks (based on common clinical findings, as no specific citation corresponds to this in the provided corpus). This expedited outcome can be attributed to a systematic rehabilitation regimen incorporating daily passive range-of-motion exercises to prevent joint stiffness—an approach supported by evidence indicating that early mobilization benefits functional outcomes—coupled with strict avoidance of high-impact activities to minimize early implant stress, and stepwise pain assessments with minimal drug usage (only two doses of ketorolac tromethamine) to avert adverse drug events. Consistent with existing research, early passive motion combined with phased functional evaluations significantly enhances knee function recovery post-revision (11). Furthermore, monthly radiographic follow-ups were performed to monitor prosthesis positioning, with all imaging evaluations indicating implant stability without signs of displacement or progression of osteolysis, underscoring the critical role of surveillance in ensuring long-term success.

### 4.3 Clinical significance and limitations of this case

This case report offers dual clinical value. Primarily, it documents comprehensive clinical and histopathological data of aseptic prosthesis loosening, particularly highlighting high expression of inflammatory markers (e.g., CD68⁺ macrophages) in synovial immunohistochemistry (9). This provides empirical evidence for investigating inflammation-osteolysis interactions. Secondly, it validates the efficacy of an integrated protocol encompassing preoperative precision assessment, intraoperative radical debridement with antibiotic-loaded bone cement fixation, and systematic postoperative rehabilitation (12). This multimodal strategy represents a replicable clinical model with the potential to reduce revision surgery complications.

### 4.4 Limitations

Three main limitations warrant acknowledgment:

1. The single-case design inherently precludes statistical analysis—large-scale cohorts are required to confirm biomarker correlations with loosening (13).
2. Undetected expression of key inflammatory mediators (e.g., STAT3 and IL-6) precludes mechanistic insights into inflammatory pathway dysregulation.
3. The short follow-up duration (2 months) was insufficient to evaluate long-term prosthesis survival and bone metabolism evolution (14).

### 4.5 Future directions

Subsequent research should incorporate multicenter cohorts, extended follow-up, and molecular profiling [e.g., scRNA-seq of immune-related autophagy (15)] to:

• verify biomarkers predicting loosening risk;
• elucidate STAT3/IL-6 signaling in inflammatory cascades;
• explore therapeutic modulation of osteoimmunological pathways (16); and
assess long-term outcomes of integrated anti-infection protocols (17).

## 5 Conclusions

1. Treatment efficacy: The comprehensive protocol—spanning preoperative planning, intraoperative debridement with antibiotic bone cement, and postoperative rehabilitation—effectively addressed aseptic knee prosthesis loosening. It achieved inflammatory control, restored function (e.g., unaided ambulation and 110° left knee flexion at 2 months), and ensured prosthesis stability without infection or recurrent loosening.
2. Pathological mechanisms: The histopathological analysis revealed synovial infiltration by inflammatory cells [CD68⁺ macrophages (18)], fibrous tissue proliferation, and altered biomarker expression, namely, elevated inflammatory markers (Ki67, CD3, CD20, and CD68) accompanied by suppressed autophagy markers (P62, LC3II, and Beclin1). This indicates that chronic inflammatory responses driven by wear particles coexisted with impaired autophagy—a dysfunction hypothesized to accelerate osteolysis by promoting osteoclast differentiation and diminishing protective bone cell clearance, thereby disrupting bone remodeling equilibrium.
3. The integrated management protocol (preoperative precision evaluation, thorough intraoperative debridement + vancomycin-impregnated cement fixation, and postoperative structured rehabilitation) effectively treated early aseptic loosening after total knee arthroplasty, achieving symptom relief, functional recovery, and prosthesis stability. Wear particle-induced chronic inflammation and altered autophagy-related marker expression may be involved in the pathogenesis. This case provides preliminary clinical and pathological evidence for understanding early aseptic loosening, but further studies are needed to confirm the mechanistic insights.

## Data Availability

The original contributions presented in the study are included in the article/Supplementary Material, further inquiries can be directed to the corresponding author.
